# Yoga, sexual dysfunction, and female concussions: a mind–body pilot intervention

**DOI:** 10.3389/fneur.2026.1705668

**Published:** 2026-03-12

**Authors:** Martina Anto-Ocrah, Hannah Garth, Caroline F. Pukall, Michele D. Levine, Sally A. Sherman, Katherine Snedaker

**Affiliations:** 1 Department of Medicine, Division of General Internal Medicine, University of Pittsburgh School of Medicine, Pittsburgh, PA, United States; 2 Department of Epidemiology, Graduate School of Public Health University of Pittsburgh, Pittsburgh, PA, United States; 3 University of Rochester School of Medicine and Dentistry, Rochester, NY, United States; 4 Department of Psychology, Queen’s University, Kingston, ON, Canada; 5 Department of Psychiatry, University of Pittsburgh School of Medicine, Pittsburgh, PA, United States; 6 Physical Activity Research Center, Department of Health & Human Development, University of Pittsburgh, Pittsburgh, PA, United States; 7 PINK Concussions, Norwalk, CT, United States

**Keywords:** yoga, sexual dysfunction, mood correlates, PTSD, anxiety, depression

## Abstract

**Introduction:**

Sexual wellbeing is a key part of health and should be included in concussion recovery. While yoga can improve concussion outcomes, its effects on sexual wellbeing in women with post-concussion symptoms are unclear. We aimed to evaluate the feasibility (Aim 1) of a brain injury–tailored yoga program, and its impact on sexual function (Aim 2a) and mood correlates—depression, anxiety, PTSD (Aim 2b)—in women aged 18+. We also assessed sustainability (Aim 3) and acceptability/ participant satisfaction with the intervention (Aim 4).

**Methods:**

Participants were recruited from PINK Concussions, a social media support network for female concussion patients with symptoms lasting at least 1 month. Twelve participants were enrolled in a 6-week yoga program, and an additional 15 were assigned to a wait-list control condition. Participants received a self-report survey 1-week post- yoga intervention (week 7) and 5 weeks post-intervention (week 11). All participants were yoga novices, defined as practicing yoga once a month or less. Sessions were held over Zoom and led by a certified yoga instructor. The Female Sexual Functioning Index-6 (FSFI-6) was used to assess sexual dysfunction. Participants also completed measures of depression (PHQ-9), anxiety (GAD-7), and PTSD (PCL-5). We used difference in differences estimates to calculate the average treatment effects, and the counterfactual means of the intervention group. We also used Glass’ Delta (*Δ*) to calculate effect sizes.

**Results:**

(Aim 1) Fifty-seven eligible women expressed interest in the yoga program. Invitations were sent in two batches to limit sessions to 15 participants. For Group 1 (Yoga group), 27 women were invited; 16 (59%) confirmed interest, and 12 enrolled after exclusions due to scheduling constraints. Attendance varied, with 6/12 (50%) completing ≥3 sessions; 5 of these 6 (83%) completed both week-7 and week-11 follow-up surveys. For Group 2 (wait-list controls), 34 women were invited, including those unable to attend Group 1. 15 (44%) confirmed participation, and 11 (73%) completed both follow-up surveys. Outcomes were compared between the yoga and wait-list control groups. At 1-week post-yoga intervention (Aims 2a and 2b), participants reported substantial improvements in their sexual functioning and mood symptoms. Effect sizes: large for sexual function (*Δ* = −0.50), medium for anxiety (−0.30), small for PTSD (−0.15) and depression (0.05). At 5 weeks (Aim 3), scores declined slightly but stayed above baseline. Sexual function retained a medium effect size (−0.34). All other domains had small effect sizes. Acceptability was high, with mean satisfaction scores of 8.7/10 (Aim 4). Using open-ended text boxes, participants commented on the intervention’s impact on their mental health, physicality, social connection/isolation/belonging; and provided suggestions for improvement.

**Conclusion:**

Yoga has the potential to improve women’s sexual functioning after concussion and offers a non-pharmacological alternative for treating mood and other important correlates of women’s sexual well-being. Larger randomized trials are needed.

## Introduction

Traumatic Brain Injuries (TBIs) are common, occurring in an estimated 1.5 million people in the United States per year (1). TBIs can result in inflammation, gliotic scar formation, and hippocampus injury among many other physical effects (2). Mild TBIs, or concussions, characterize approximately 75% of TBIs in the US and can result in persistent post-concussion symptoms (PPCS): prolonged symptomatology resulting from concussions or post-concussion symptoms (PCS), which can last for months, at times years, for about 25% of patients (3, 4). This group of patients are mostly women, and their long-term outcomes tend to be worse than men’s (5–11). They also tend to experience more severe mood disorders, like anxiety, depression, and Post Traumatic Stress Disorder (PTSD) (12–19). The effect of concussion on women’s sexual wellbeing is also emerging (11, 20–22).

Sexual Dysfunction (SD) is a distressing clinical condition characterized by changes in sexual response and sexual pleasure, including low desire/arousal, difficulty achieving orgasm, and painful intercourse (23). SD is an imperative consideration in health according to the World Health Organization, specifically in its role in human survival and enrichment of life (11, 24). SD can lead to negative impacts on quality of life, specifically on physical and emotional satisfaction, and it is often associated with negative relationship outcomes (25–28). One study shows that 61% of women with concussions experience SD in the acute post-injury phase (6–10 weeks), compared to only 40% of extremity injured controls (*p* = 0.03) (21). Women have also been shown to report significantly more SD than men with TBI (11). Additionally, a study showed that women with concussions and SD are eight times more likely to have increased depressive and anxiety symptoms, and also experienced nearly six times the burden of PPCS compared to the control group (21). SD also interacts with psychosocial issues like mood disorders. For example a bidirectional association of SD and depression has been established and there is substantial comorbidity between SD, PTSD, depression, and anxiety (29–31). Hypotheses for SD after TBI include negative impacts on the HPG axis (32–36). These hypotheses link TBIs to decreasing levels of LH and FSH, two gonadotropins that control secretion of estrogen and progesterone, the female sex hormones (32). Estrogen and progesterone are integral for vaginal sensation and sexual arousal, and for mediating low sexual arousal respectively, so interruptions in their secretion leads to vaginal lubrication and sexual arousal issues (33–36). Sexual wellbeing is an important aspect of health and should be considered in the recovery paradigm of female patients with PPCS. Including sexual wellbeing in rehabilitation efforts in the treatment of women after concussion is imperative to decreasing the public health burden and improving the clinical care of women.

The treatment of SD following concussion in women should complement other concussion treatment modalities, and integrate medical, psychological, and physical rehabilitation strategies. A transdisciplinary approach might be the best way to improve SD following head injury (37). Yoga, a low-impact, complementary practice that encompasses physical postures combined with meditation and breathing techniques, has been used as a non-pharmacological treatment option for female SD in healthy non-head-injured populations (38). A systematic review and meta-analysis examining the effects of yoga on sexual function in healthy adults without head injuries found that yoga interventions led to a statistically significant improvement in sexual function compared to control groups across 10 randomized controlled trials, with the most notable benefits observed in women (effect size = −0.36; 95% CI: −0.52 to −0.21, *p* < 0.00001), particularly in the areas of arousal, lubrication, and orgasm (39). Yoga has also been shown to improve several PPCS including sleep, cognition, pain, and mood (depression, anxiety, PTSD) symptoms, important correlates of sexuality (40, 41). Additionally MRI, fMRI, and SPECT studies show the promising effect of Yoga to lessen neurodegenerative declines (42–45). However, no studies, to these authors’ knowledge, have been done on the use of yoga to treat female SD after concussion (21).

In this pilot study, we evaluated the feasibility (Aim 1) of a brain injury–tailored yoga program in order to determine the practicality of delivering the intervention as intended, including recruitment and retention. We then tested the effectiveness of the six-week yoga intervention to treat female SD in concussion patients aged 18 and older. First, we asked the question “Is yoga efficacious in treating SD and mood correlates in female concussion patients?” We hypothesized that compared to wait-listed controls, the yoga group would have reduced SD [i.e., higher scores on the Female Sexual Functioning Index-6 (FSFI-6) at 1 week post-intervention (Aim 2a)]. We also hypothesized that mood scores [depression, anxiety, post-traumatic stress disorder (PTSD)], which are important correlates of sexual wellbeing, would improve at 1 week post-intervention (Aim 2b) (21). Secondly, we asked “How sustainable is the effect of intervention?” We hypothesized that the yoga group will continue to have reduced SD and mood scores at 5 weeks post-intervention (Aim 3). We also asked “How satisfied were patients with the intervention?” To evaluate this, we used Likert scales and open-ended prompts to ask participants about their experiences with the intervention (Aim 4).

## Methods

### Participants

This study was conducted in collaboration with PINK Concussions, a nonprofit organization dedicated to supporting women and girls experiencing persistent post-concussion symptoms (PPCS) resulting from mild traumatic brain injuries (mTBI) caused by sports, military service, motor vehicle accidents, domestic violence, and other events. The organization provides online peer support to a community of over 40,000 individuals through platforms such as Facebook, Instagram, Twitter, and TikTok. Women dealing with concussion symptoms lasting longer than a month connect with PINK for additional social and emotional support (46).

### The yoga program

The LoveYourBrain (LYB) Yoga program is a six-week, evidence-based, light intensity yoga and meditation curriculum developed specifically for individuals living with traumatic brain injury (TBI) (47–50). Delivered online and synchronously, the program is led by experienced instructors with more than a decade of teaching experience in yoga. The instructors followed the standardized LYB protocol (47–50). Each weekly session runs for 75 min and combines gentle postures, guided breathing exercises (pranayama), yoga nidra (deep relaxation), and meditation practices. These elements are thoughtfully adapted to accommodate the needs and physical abilities of people with TBI, allowing participants to modify movements based on their comfort and limitations. TBI specific considerations included postures facing the camera and adaptations for those with balance and/or vestibular challenges as well as other co-morbidities. These poses offered adaptations without inversions, with chairs as support, and with slow, repetitive instructions. Before each of the yoga sessions, participants are e-mailed prerecorded mindfulness tools that focused on a specific theme for the week – resilience, mindfulness, intentions, realistic optimism, positive thinking, and gratitude—to foster a growth (vs fixed) mindset, so participants are better able to transform perceived challenges into catalysts for learning and growth. The online sessions were conducted on Wednesdays from 12–1:15 p.m. (Eastern standard time). Each group was intentionally limited to 10–15 participants to create a manageable size that promotes safety, supports active engagement, and allows instructors to provide personalized attention and guidance tailored to each participant’s needs throughout the session.

### Recruitment and approach

Between November 10th to 13th 2023, participants received an IRB-approved REDCap survey link to invite women in PINK Concussions to participate in the yoga study. Upon clicking the survey invitation link, survey takers were brought to the IRB-approved consent page, which listed the risks, benefits, incentives, and overall research processes. After reviewing the consent page, participants were prompted to consent “yes” in order to move on to the survey body. Those who did not consent were sent to a “Thank you page” which exited them out of the study. To be eligible, participants needed to be (1) 18 years of age or older, (2) assigned female at birth, (3) be a yoga novice—defined as practicing yoga once a month or less—and (4) be a United States resident (as specified by the IRB). Focusing on yoga novices allows us to evaluate the feasibility, acceptability, and initial learning curve of the intervention among those with no prior exposure. This is particularly important when determining whether the program is accessible and engaging for beginners, who represent a key target population for many yoga-based interventions.

To ensure that we had no more than 15 participants per session, we sent follow-up email invitations to interested participants in two batches from 11/22/2023 to 11/30/2023. The first cohort was assigned to the Group 1 Yoga session which ran from Nov 29th to Jan 3rd. Those who were unable to participate were confirmed and “wait listed” for the Group 2 Yoga session which ran from Feb 28th to April 3rd. The latter group was enlisted as wait list controls and sent the week 7 and week 11 surveys to compare to the yoga group. A wait-list control group was used in this study. That is participants assigned to the wait-list control condition did not receive the intervention during the study period and were offered the intervention only after completing study assessments. This approach allows for a comparison between those receiving the intervention immediately and those who have not yet participated, while ensuring that all participants eventually have access to the potentially beneficial program. Wait-list controls are commonly used in behavioral and clinical research, as they provide an ethical and practical method for evaluating intervention effects (51, 52).

### Survey measures

Participants provided basic demographic (age, race, marital status, education, etc.) and concussion-related (time since injury, mechanism, duration of PINK membership, concussion history, etc.) information. The Female Sexual Functioning Index-6 (FSFI-6) was used to assess sexual dysfunction. The FSFI-6 is a validated questionnaire that uses a numerical scoring system of 2.0–30, with a higher total score indicating higher sexual function (53). It has been used to evaluate changes in sexual functioning after yoga in healthy populations of women (39). Assessments of sexual dysfunction were conducted at baseline, 1-week post-yoga intervention (week 7 and week 5 weeks post-intervention (week 11)). Depression (PHQ-9), a known correlate of sexual dysfunction, was also examined, as were anxiety (GAD-7) and PTSD (PCL-5). The PHQ-9 is a validated assessment of depression, with a numerical scoring system of 0–27 (54). Higher scores indicate higher levels of depressive symptoms. Cutoffs are 0–4: Minimal depression, 5–9: Mild depression,10–14: Moderate depression, 15–19: Moderately severe depression, 20–27: Severe depression. The GAD-7 is a validated questionnaire that assesses anxiety, with a score range of 0–21, with higher scores indicating higher levels of anxiety (55). Cutoffs are: 0–4: minimal anxiety, 5–9: mild anxiety, 10–14: moderate anxiety, 15–21: severe anxiety.

The PCL-5 screens people for PTSD using a numerical scoring system with a range of 0–80, with higher scores indicating higher incidence of PTSD symptoms (56). A cut-point score of 31–33 is used to indicate a probable diagnosis of PTSD.

### Data analyses

Symptoms rated by the yoga group were compared to those of the wait-listed group at 1 week post intervention (week 7) and 5 weeks post intervention (week 11). We used difference in difference (DID) estimates to calculate the Average Treatment Effects (the difference between the average outcome of a group that received treatment and the average outcome of the control group). DID approaches are one of the most popular quasi-experimental analytical approaches used in research particularly when randomization is not feasible. DID approaches allow for estimation of treatment effect dynamics before and after treatment, and provides a robustness check for observed parallel trends (57). The DID method compares changes in an outcome over time between an intervention and control group and is thus ideal for estimating the effects of interventions that are applied at the group level rather than at the individual level. The use of difference in difference analyses inherently controls for time-invariant confounding, meaning factors that differ between treatment and control groups, *and* do not change over time. For example: baseline demographic differences (age, sex, education level) or pre-existing health conditions (e.g., time since injury, history or previous concussions). These factors are differenced out because DID looks at changes over time within groups and compares those changes between groups (58, 59).

We also used counterfactual mean calculations to determine the expected scores of the intervention group, assuming the slopes of the control group (60). The counterfactual mean assesses the true impact of the yoga intervention by answering the question “If not for yoga, what would the intervention group’s mean be, assuming the same slope as control group?”

Finally, Glass’s delta (Δ) was used to calculate effect sizes. Glass’s delta quantifies the standardized difference between the means of the treatment and control groups, using the standard deviation of the control group for standardization. The following cutoffs are used to determine the magnitude of the effect:

- Small effect size: Δ ≤ 0.20
- Medium effect size: 0.20 < Δ < 0.50
- Large effect size: Δ ≥ 0.50

A positive value suggests the experimental group’s mean is higher than the control group’s, while a negative value indicates the opposite (direction).

Missing Data: Missing data rates fell between 20 and 50%, a range in which imputation is generally discouraged, particularly for exploratory research (61). In addition, missingness was largely not at random, as participants frequently reported difficulty attending sessions scheduled during working hours, especially among those who were employed. Under these conditions, imputing missing outcomes would likely introduce substantial bias. Therefore, analyses were conducted without imputation (61).

## Results

### Aim 1: Feasibility

Fifty-seven women who met the eligibility criteria expressed interest in the yoga program. To ensure a maximum of 15 participants per session, email invitations were sent to interested participants in two batches from 11/22/2023 to 11/30/2023.

Group 1 Yoga session (Nov 29th to Jan 3rd): Invitations were sent to 27 randomly selected participants; 16 responded to confirm interest (59.3%); however, 4 were unable to attend the sessions because of the time commitment, of work schedules, and of time zone differences. All 4 were invited to participate in the wait-list control group. Therefore, 12 of the interested 16 were confirmed for the first yoga group (75%). Six of the 12 attended zero sessions, 1 completed 3 sessions, 1 completed 5, and 4 completed all 6 sessions. The Week 7 follow-up survey was sent to those who completed at least 3 yoga sessions (n = 6/12 (50%)), and 5 of the 6 (83%) completed both the week 7 and week 11 follow-up surveys.

**Figure 1:**
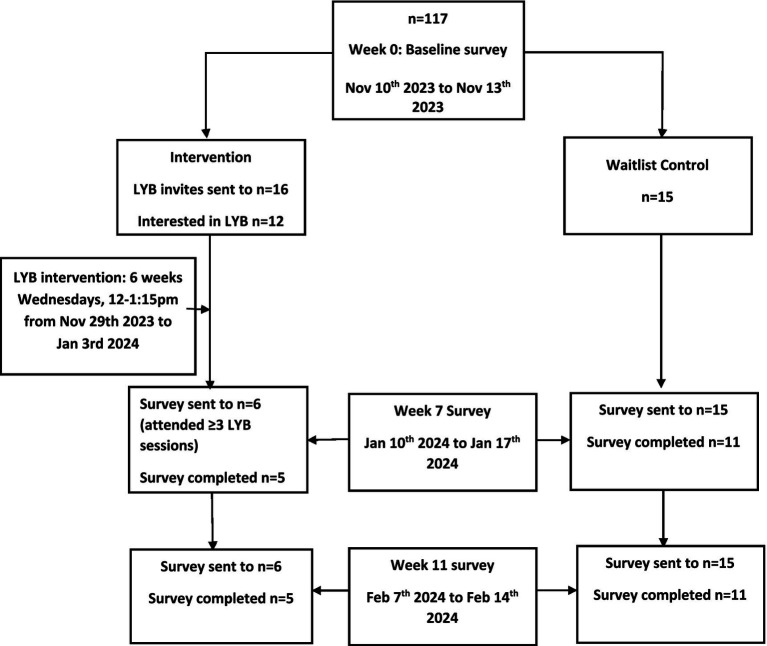
Here flowchart of participants enrolled at each stage of the study.

Group 2 Yoga session (Feb 28th to April 3rd), wait list control group: Invitations were sent to the remaining 30 plus the 4 who were unable to attend the first session, and 15/34 (44%) confirmed interest and were put in the wait-list control group. These participants were sent the week 7 and week 11 surveys; 11 of the 15 (73%) completed both surveys and scores on the measures were compared to those of the yoga group (Figure 1).

As shown in Table 1, most of the participants had been members of the PINK Facebook group for at least 1 year (4/5 yoga group and 11/11 waitlist controls). All 5 of the yoga group participants identified as white/Caucasian with mostly advanced degrees (i.e., Master’s/advanced/terminal degrees), additional schooling after a bachelors. Comparatively, the waitlist control group was made up of 3 African American/black women and 8 white/Caucasian women, with a range of educational attainment. A majority reported being in relationships, with everyone who responded identifying as monogamous.

**Table 1:** Baseline demographic, relational, and injury-related attributes of yoga and control participants (*n* = 17; 5 = Yoga, 11 = Control).

Attributes	Yoga group (n = 5)	Wait-list controls (*n* = 11)
PINK attributes		
How long have you been a member of the PINK Facebook groups?		
Less than 1 year	1 (20%)	0 (0%)
1–2 years	1 (20%)	6 (54.55%)
3–5 years	2 (40%)	4 (36.36%)
Over 5 years	1 (20%)	1 (9.09%)
Demographic attributes		
Age: Mean ± Std Dev	50.8 ± 15.69	37.27 ± 10.21
Min, Max	22, 67	24, 53
Would you describe yourself as.?		
African American/Black	0 (0%)	3 (27.27%)
White/Caucasian	5 (100%)	8 (72.73%)
Are you Hispanic/Latino Ethnicity?	0 (0%)	0 (0%)
What is your highest level of education completed?		
Less than high school	0 (0%)	0 (0%)
High school diploma/GED	1 (20%)	1 (9.09%)
Technical training/associate’s degree	0 (0%)	2 (18.18%)
Some college/bachelor’s degree	0 (0%)	3 (27.27%)
Master’s/advanced/terminal degrees	4 (80%)	5 (45.45%)
Other (specify)	0 (0%)	0 (0%)
What is your average household income?		
Less than $30,000	0 (0%)	2 (18.18%)
$30,000–$59,999	1 (20%)	3 (27.27%)
$60,000–100,000	2 (40%)	4 (36.36%)
More than $100,000	1 (20%)	2 (18.18%)
Decline to answer	1 (20%)	0 (0%)
Parity and relationship attributes		
How many children have you had?		
Mean ± std dev	0.8 ± 0.75	1.18 ± 1.11
Min, Max	0, 2	0, 3
How many children under the age of 18 are in your household?		
Mean ± std dev	0.2 ± 0.4	0.73 ± 0.75
Min, Max	0, 1	0, 2
What is your romantic relationship status?		
Single and not in a relationship	1 (20%)	1 (9.09%)
In a relationship but not married	1 (20%)	6 (54.55%)
Married	2 (40%)	4 (36.36%)
Divorced or separated	1 (20%)	0 (0%)
When it comes to romantic relationships, do you prefer …	n = 5	n = 11
Men	3 (60%)	11 (100%)
Women	1 (20%)	0 (0%)
Missing	1 (20%)	0 (0%)
Would you describe yourself as?	n = 5	n = 11
Monogamous (having a romantic/sexual relationship with one partner at a time)	4 (80%)	11 (100%)
Missing	1 (20%)	0 (0%)
Injury attributes		
When was your most recent concussion?		
≤1–3 months ago	0 (0%)	1 (9.09%)
4–11 months ago	3 (60%)	4 (36.36%)
1–2 years ago	0 (0%)	3 (27.27%)
3–5 years ago	0 (0%)	2 (18.18%)
>5 years ago	2 (40%)	1 (9.09%)
How did you sustain your most recent concussion?		
Assault	0 (0%)	1 (9.09%)
Fall	1 (20%)	1 (9.09%)
Motor vehicle accident	0 (0%)	5 (45.45%)
Non-fall sports injury	1 (20%)	0 (0%)
Recreational vehicle accident	0 (0%)	1 (9.09%)
Struck by motor vehicle	0 (0%)	1 (9.09%)
Struck by object	1 (20%)	1 (9.09%)
Other mechanism	2 (40%)	1 (9.09%)
Did you have a history of concussions before your most recent one?		
Yes (ask next question)	4 (80%)	5 (45.45%)
No (skip next question)	1 (20%)	6 (54.55%)
How many previous concussions did you have before the most recent one?	n = 4	n = 5
Mean ± std dev.	3 ± 1.22	2 ± 0.63
Min, Max	2, 5	1, 3
Baseline sexual function & psychiatric comorbidities (Mean ± Std Dev)		
Sexual function (FSFI- 6)	11 ± 10.77	19.15 ± 9.78
Depression (PHQ-9)	9.5 ± 4.53	9.2 ± 5.5
Anxiety (GAD-7)	7.67 ± 3.56	7.33 ± 3.38
PTSD (PCL-5)	28.16 ± 24.30	26.73 ± 18.58

Concussion demographics varied between groups. All 5 yoga participants had their most recent concussion more than 4 months ago, with 2/5 occurring over 5 years ago. The waitlist control group had more varied concussion timelines. The etiology of their most recent concussion varied between groups, including sports-related and struck by object in the yoga group and assault and motor vehicle accidents in the waitlist control group. The yoga group was more likely to have a history of multiple concussions: 4/5 had sustained more than one concussion in the past, with a mean of 3 (±1.22) (Std Dev) historic concussions compared to a mean of 2 (±0.63) (Std Dev) for the waitlist control group.

### Aims 2a and 2b: Efficacy of yoga intervention in treating SD and mood correlates

**Figure 2:**
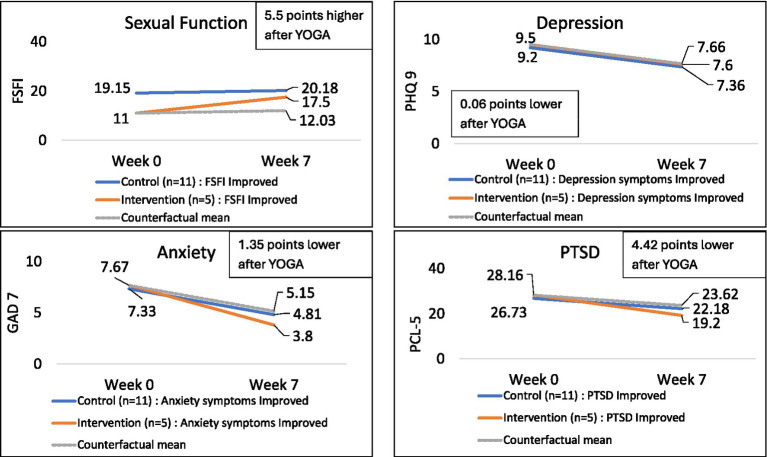
Week 7 (1-week post-intervention) mean scores and counterfactual mean calculations for the yoga intervention group and control group (*n* = 17; 5 = Yoga, 11 = Control).

As shown in Figure 2 (see Supplementary Tables), FSFI-6 scores increased from 11 at baseline to 17.5 post-yoga, a 6.5-point increase compared to the 1.03-point increase for the control group, a treatment effect of 5.5 points. The yoga intervention participants started with higher anxiety scores on the GAD-7, but these scores decreased and improved, surpassing the control group with an average treatment effect of −1.35 points on the GAD-7. PTSD scores followed a similar trend, with an average treatment effect of −4.42 points. Depression scores (PHQ-9) also decreased but to a smaller degree (−0.06) (Figure 2; Table 2).

**Table 2:** Treatment effect and counterfactual means to answer the question “If not for yoga, what would the intervention group’s mean be, at 1-week post-intervention assuming same slope as control group?” (*n* = 17; 5 = Yoga, 11 = Control).

Analysis	FSFI-6	PHQ 9	GAD 7	PTSD
Treatment effect (difference in difference)	5.47	−0.06	−1.35	−4.42
Counterfactual mean of yoga group	12.03	7.66	5.15	23.62
Actual mean of yoga group	17.50	7.60	3.80	19.20

In all domains, the actual mean of the intervention group was more improved than the expected, counterfactual mean (Table 2). Without the yoga intervention, the expected FSFI-6 mean scores 1 week post-intervention would be 12.0 vs. the actual, observed mean score of 17.5; GAD-7 expected scores for the yoga group would be 5.2 vs. the actual mean score of 3.8; expected PHQ-9 scores would be 7.7 vs. the actual mean of 7.6, and the expected PTSD scores would be 23.6 vs. the actual mean of 19.2.

As shown in Table 3, at 1-week post-intervention, the yoga intervention had the largest impact on sexual functioning (−0.50) and anxiety (−0.30), which had large and medium effect sizes (*Δ*) respectively.

**Table 3:** Effect sizes.

Glass’ D and week	FSFI-6	PHQ 9	GAD 7	PTSD
Glass’ D (Δ) Week 7 (1- week post-intervention)	−0.50 (large effect)	0.05 (small effect)	−0.30 (medium effect)	−0.15 (small effect)
Glass’ D (Δ) Week 11 (5 weeks post-intervention)	−0.34 (medium effect)	0.2 (small effect)	−0.02 (small effect)	−0.13 (small effect)

### Aim 3: Sustainability of the yoga intervention

**Figure 3:**
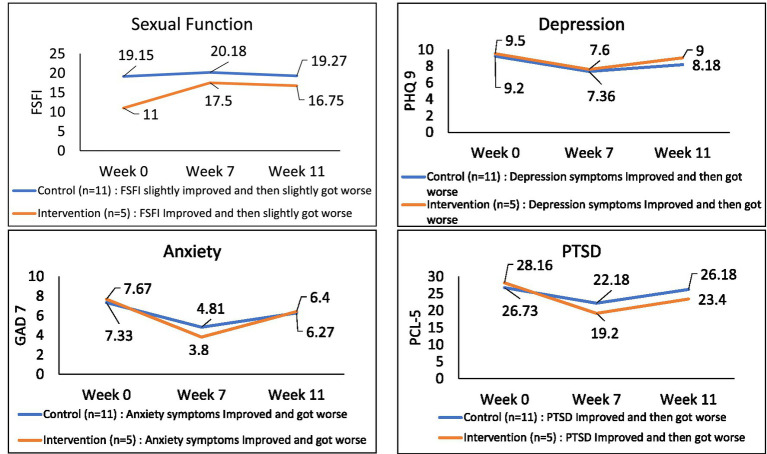
Week 11 (5 weeks post-intervention) trends compared to week 7 (1 week post) (*n* = 17; 5 = Yoga, 11 = Control).

Follow-up surveys were again completed at 5 weeks post-intervention to assess the sustainability of the response to the yoga intervention (Figure 3; see Supplementary Tables). At this timepoint, FSFI scores declined slightly to 16.75 (from 17.5 at week 7), but remained drastically improved from baseline scores. Depression (PHQ-9) and anxiety (GAD-7) scores also increased, but none returned to pre-yoga, baseline levels. Sexual functioning remained the most impacted, with medium effect sizes (−0.34). All other domains had small effect sizes (Table 3).

### Aim 4: Acceptability-participant satisfaction with intervention

On a 10-point Likert scale asking participants about their satisfaction with the intervention (0 = extremely dissatisfied and 10 = extremely satisfied), the mean score was 8.7 (±2.2 Std Dev; range 4,10), and all replied that they would recommend the LYB program to others in their network.

Using open-ended text boxes, we asked the yoga participants to describe their experiences with the intervention. Responses were reviewed using a brief thematic analysis approach, in which two study team members (HG and MAO) independently read the comments, identified recurring ideas, and grouped them into themes. Participant comments corroborated the data. Most of the comments were positive, and specific themes included physical changes, social support, and interest in more frequent sessions. Suggestions for improvement focused on increasing the frequency of the intervention.

### Comments related to mood and mental health

“I have been more productive since I started the program. I am able to filter out the things out of my control, just focus on today and doing what I can. I am not shutting down when I am overwhelmed. I am just doing my best to get things done. If I do not get to it today, it will be there tomorrow. This attitude shift has been so helpful.” (Participant #12).

“Talking about my [TBI] experience causes me to feel very emotional. It was hard for me to settle in the beginning because being in my body felt scary. I think the yoga classes helped with that.” (Participant #175).

### Comments related to physicality

“I can lift my arms about my head! My PT could not believe it! I had to start out modifying all the arm movements, but it gradually got easier to do. I have not been able to lift my arms about my head, without causing extreme spasms all night, for years” (Participant #12).

“Feeling more relaxed” (Participant #37).

### Comments related to the helpfulness of the yoga program and the social connection/isolation/belonging

“I LOVED being able to be with others who also had brain injuries. It was so powerful. I did not feel so alone. WOW!” (Participant #175).

“Sharing common experiences and learning that I am not the only one who has experienced these difficulties. Seeing how other people are coping with issues from TBI.” (Participant #37).

“Being able to connect with other people with brain injuries. It was the first interaction I have had with people with TBI” (Participant #375).

“I am more committed to my self care than caring for or trying to love someone who does not want to treat me right. I want to treat myself right and I am more OK with standing up for my needs than letting him decide who I am.” (Participant #175).

### Suggestions for improvement

“It was amazing meeting and talking with people who understand. I would have liked these conversations to be longer.” (Participant #375).

“I wish it met twice a week or once a week for 8 weeks, instead of 6” (Participant #12).

“But I think at the beginning I really just needed someone to HEAR ME, SEE ME. I mean they all did, but I was craving more. One time per week did not feel like enough. I mean it was and I am so thankful but I think I just wanted more!” (Participant #175).

## Discussion

To our knowledge, this is the first study to examine the efficacy of yoga on SD and mood correlates in females with concussions (21). This study is a pilot, preliminary, and exploratory investigation primarily designed to evaluate the feasibility, efficacy, and acceptability of a yoga intervention for sexual dysfunction after concussion. Our primary goal was to assess whether participants could engage with and tolerate the intervention, and to identify potential barriers or facilitators for future larger-scale studies. Whereas other research groups have addressed yoga’s effect on SD and TBI recovery, in this study, we evaluated the intersection of all three factors (Yoga, SD, and TBI) by assessing the impact of yoga on SD after concussion/mild TBI (39–41, 46–50). Feasibility (Aim 1) of the yoga intervention was supported by strong initial interest and acceptable retention among enrolled participants. Fifty-seven eligible women expressed interest, and enrollment targets were met through staggered recruitment. While scheduling constraints limited enrollment and session attendance in the intervention group, half of enrolled participants completed at least three sessions, and follow-up survey completion was high among those meeting this threshold (83%). Participation and retention in the wait-list control group were similarly strong, with nearly three-quarters completing follow-up assessments. These findings suggest that recruitment, enrollment, and outcome assessment procedures were feasible, although session attendance may be improved in future studies through increased scheduling flexibility. Our findings show that Yoga might be efficacious in treating female SD and mood correlates (Aims 2a and 2b); the treatment effects are sustainable (Aim 3); and patients are not only satisfied with the intervention, but they want more of it (Aim 4). Our study’s success in producing these results as well as it’s ability to be expanded and replicated due to the use of online communities and classes show promise, and demonstrate that mind–body practices like yoga could be efficacious in the treatment of SD and mood correlates, specifically depression, anxiety, and PTSD in women after concussion.

After 6 weeks of yoga, participants showed improvements in all domains compared to the controls. At 1-week post-intervention, both SD and mood—particularly anxiety—had improved substantially. These improvements remained above baseline levels at 5 weeks post-yoga, and sexual functioning scores especially remained robust in effect size. The connection between the physical and psychological is complex, but treatments that strengthen both, like yoga, can be helpful in improving people’s quality of life and pain (47). This mind–body connection is perhaps even more important in treating people with TBI, because their emotional and physical brain are so intricately connected (62). There were varying comments on physical improvement after intervention by participants. One explained that she could now lift her hands above her head, something she was unable to do before. The intervention strengthened her physical post-concussion symptoms. Strength and a lack of fatigue may allow some people to explore their sexuality and increase the frequency in which they engage in sexual activity (20). So, while yoga could help with psychological aspects of sexuality, it also might help with strength and therefore physicality in sex. Further research could focus on the physical improvements and/or challenges that women with SD after concussion experience with consistent yoga practice.

Other comments on the intervention included appreciation of the shared experiences and community among participants, one mentioning she “did not feel so alone.” A couple of participants wanted to meet more than once a week to foster that feeling of community because they valued the discussions. These comments suggest that social support might also be involved in the improved outcomes. Social support, especially with those who have similar experiences, can lead to improved sexual function, mood, and anxiety, further explaining the improved scores observed (63–70). Research has consistently shown that social support can positively impact both psychological and physiological outcomes. For individuals experiencing sexual dysfunction, depression, or anxiety—particularly in chronic disease contexts such as PPCS—being part of a supportive community can enhance emotional well-being and increase treatment efficacy. Social connection has been linked to improvements in sexual function, reductions in depressive symptoms, and lower levels of anxiety (63–70). Support groups that involve shared lived experiences, especially, provide emotional validation, normalization of symptoms, and coping strategies that may not emerge in individual treatment settings in TBI rehabilitation. Studies show that social support-whether in person or online, is one the strongest predictors of TBI recovery (68–70). In the context of group interventions for women’s sexual health and emotional well-being, peer support can be particularly impactful. Studies by Bossio et al. evaluating the impact of Mindfulness-Based Group Therapy- and Meston et al.-assessing the effects of expressive writing on sexual dysfunction and mood- show that women who engage in group-based therapies or support programs often report enhanced intimacy, communication, and emotional safety, which can translate into better sexual satisfaction and function (67, 71). Thus, the participants’ appreciation of the communal aspects of the program suggests that social connection and mutual support were key therapeutic elements. This added layer of support may help explain the improved outcomes noted in measures of sexual function, mood, and anxiety.

Mindfulness is an important part of the yoga practice and meditation, and group mindfulness therapy has been shown to improve sexual wellness (63, 64, 72). It is possible that more purposeful integration of mindfulness techniques could further support the mood and SD improvements. We hypothesize that another mechanism by which yoga helped improve outcomes in the intervention group could be in the alteration of the levels of biomarkers. Meditation is an integral part of the practice of yoga and is a technique in which someone focuses on an object or activity to train their mindfulness and awareness (73). Meditation has been linked to decreased pro-inflammatory markers and increased anti-inflammatory markers (74). Meditation has physical effects such as sympathetic nervous system deactivation, decreased systolic blood pressure, decreased heart rate, less heart rate variability, and decreased cortisol (74). It also has psychological effects such as higher levels of emotional acceptance, described as a lower emotional response to adverse events, less anxiety, and increased emotional intelligence (75–77). These psychological effects could be explained by meditation’s effects on levels of neurotransmitters. It increases serotonin, a neurotransmitter that, when low, is implicated in depression (77). It increases levels of gamma-aminobutyric acid (GABA), a neurotransmitter that, when low, increases symptoms of panic disorder and PTSD (78). It also decreases levels of norepinephrine, which is a neurotransmitter implicated in anxiety (77). While we do not measure these biomarkers in this study, we do think it is important to tie these effects we are seeing into the existing research on meditation. Looking at biomarkers in yoga in this population is a possible avenue for future research. Further research might explore biomarkers like cortisol or neurotransmitter levels before and after the yoga sessions in those with concussion and SD to better evaluate mechanistic pathways.

Despite its centrality to health, many people with SD feel uncomfortable discussing their issues with providers, and women have a lower likelihood of bringing up sexual health concerns with their providers, than men (79). Discussion of sexual health in appointments with women, including sexual behavior, vaginal dryness, and libido, often bring feelings of embarrassment, fear of judgment, or modesty concerns (79). Patients express that they would feel more comfortable with sexual health discussions if their providers initiated discussion of the topic. In a pre-survey we gave PINK participants, one participant said that they had never been asked about their sexuality in an appointment with a provider, and another said it was only addressed in a pre-appointment check list and never brought up again. In post surveys, these participants commonly expressed that there were community and comfort in these yoga sessions. They felt heard and seen. We hypothesize that this online community of people with shared experiences allowed participants to more comfortably open up about their struggles with PPCS, including SD. To reduce discomfort, professionals in the fields of sex and sexuality, such as psychologists, OBGYNs, sex and rehabilitation therapists, and other professionals should be integrated on online platforms, providing evidence based information, fact checking the advice on the platforms, and possibly even offering classes or open discussions (80). However, online mind–body treatments for SD like the LYB intervention tested in this study, have advantages and disadvantages. For example, online programming might be more appealing to women, as they can be anonymized. Participants can turn off their video cameras or communicate via chat should they feel uncomfortable revealing their identities and/or discussing intimate details of their sexuality. While anonymity is easier to achieve online, there might be accessibility issues in the online programming due to timing and Wi-Fi access. To further increase accessibility of programs such as LYB, future work might provide alternative times for the online sessions, as many working individuals do not have access to 75 min of leisurely time during their workday. Studies could explore shorter sessions (~30 min) or even weekend sessions which would allow better flexibility for participation. Interestingly, the participants in our study requested longer sessions with the intervention; an observation that was supported by the sustainability findings in Aim 2. While the improvements in SD and mood remained above baseline 5 weeks post-intervention, they all did decrease slightly. These decreases could suggest that more long-term yoga classes might be beneficial for continued improvement. This could take the form of self-directed sessions or “homework” where participants can continue to practice yoga on their own after each weekly session. Xue et al. show that after a four-week online mindfulness intervention for treating women’s SD, the majority of participants completed assigned homework and felt the intervention enhanced their sexual well-being (81). Research is needed to evaluate homework adherence and impact on women’s SD in TBI contexts.

Regardless, our results show that a mind–body, meditative, mindful practice, like yoga, is a promising intervention that might improve SD and mood correlates in female concussion patients, providing impetus for a multidisciplinary, biopsychosocial treatment paradigm for these patients.

### Strengths and limitations

To our knowledge, this is the first study that examines the impact of yoga on SD in women with concussions. While observed changes in outcomes are encouraging, we cannot definitively conclude that they are due to the intervention alone. Future studies with larger sample sizes and rigorous designs—including randomization and control for confounders—will be necessary to formally assess efficacy and causal effects. Our study was impacted by small sample sizes, limiting the statistical power of our findings. High attrition may introduce attrition bias if those who dropped out differed systematically from those who completed the intervention. While this is an inherent limitation of this exploratory, preliminary study, the findings still provide useful insights into feasibility, acceptability, and potential barriers to participation, which can inform the design of larger future trials. Although such small sample sizes are not uncommon in the neurosciences, our pilot work should provide impetus for larger and more representative randomized clinical trials in the future (82, 83). Study participants were recruited from an online forum, which limits the generalizability of study findings to female concussion patients with access to online social support groups. Future research should attempt to recruit a more representative population of concussion survivors with PPCS, particularly those with SD to validate our findings. Lastly, our findings are not generalizable beyond the United States of America. While TBI is common globally, cultural expectations and experiences with sexual behavior vary significantly across countries and regions, potentially influencing how individuals perceive, experience, and report changes in sexual functioning. A more representative sample of racial and ethnic groups might also be important for similar reasons. Health care systems, provider training, social norms, and openness around discussing sexual health also differ, which may affect both the delivery of care and the willingness of patients to engage in such discussions. As a result, further research is needed to explore these dynamics in diverse cultural contexts. Another limitation which may have been introduced because we did not blind participants is expectation bias, which occurs when participants’ knowledge about receiving—or not receiving—an intervention influences their responses, potentially confounding the observed effects. In wait-list control designs, participants know they will eventually receive the intervention, which can shape their expectations and behaviors during the waiting period. Future research should use blinding techniques to mitigate this bias. Sample sizes should be large enough to address the limitations raised, as well as randomization. Lastly, as noted, this study is a pilot, preliminary, and exploratory investigation primarily designed to evaluate the feasibility, acceptability, and efficacy of a yoga intervention for sexual dysfunction after concussion. Our primary goal was to assess whether participants could engage with and tolerate the intervention, and to identify potential barriers or facilitators for future larger-scale studies. While observed changes in outcomes are encouraging, we cannot definitively conclude that they are due to the intervention alone. One reason being that time-varying factors (e.g., new medications started by only one group or changes in lifestyle that differ between groups) are not adjusted for in DID analyses and require regression models (e.g., by including covariates) which were beyond the scope of this pilot study (57). Additionally, psychiatric or medical co-morbidities of these patients at baseline, which could be potential effect modifiers, are important. Future studies with larger sample sizes and rigorous designs—including randomization, control for confounders, explore effect modification, etc. will be necessary to formally assess causal effects. Despite these limitations, our findings indicate that a mind–body intervention such as yoga—emphasizing meditative and mindful practices—can improve sexual dysfunction and associated mood symptoms in female patients with concussion. They set the stage for larger, randomized trials.

## Conclusion

A yoga practice tailored to people with TBI might positively impact SD, anxiety, depression, and PTSD in women with mild TBI. Increasing the frequency of intervention could potentially lead to longer, sustained outcomes. The practice of yoga may be a promising a non-pharmacological intervention to help women with SD and mood correlates after concussion.

## Data Availability

The original contributions presented in the study are included in the article/Supplementary material, further inquiries can be directed to the corresponding author.
