# Supplementary material for: Yoga, sexual dysfunction, and female concussions: a mind–body pilot intervention
Source: Front Neurol. 2026 Mar 12;17:1705668. doi: 10.3389/fneur.2026.1705668 (PMC13019368; doi:10.3389/fneur.2026.1705668)
Supplement: Supplementary file 1 [file Table_1.docx]

**FSFI scores:**

|  | **Group** | **Baseline** | **Week7** | **Week11** |
| --- | --- | --- | --- | --- |
| Control Group | Mean | 19.15 | 20.18 | 19.27 |
|  | STD | 9.78 | 5.53 | 7.70 |
|  |  |  |  |  |
| Intervention Group | Mean | 11 | 17.5 | 16.75 |
|  | STD | 10.77 | 10.84 | 10.81 |
|  |  |  |  |  |

**PHQ-9 scores**

|  | **Group** | **Baseline** | **Week7** | **Week11** |
| --- | --- | --- | --- | --- |
| Control Group | Mean | 9.2 | 7.36 | 8.18 |
|  | STD | 5.50 | 4.90 | 4.71 |
|  |  |  |  |  |
| Intervention Group | Mean | 9.5 | 7.6 | 9.0 |
|  | STD | 4.53 | 5.40 | 5.48 |

**GAD-7 Scores**

|  | **Group** | **Baseline** | **Week7** | **Week11** |
| --- | --- | --- | --- | --- |
| Control Group | Mean | 7.33 | 4.81 | 6.3 |
|  | STD | 3.38 | 3.50 | 4.52 |
|  |  |  |  |  |
| Intervention Group | Mean | 7.67 | 3.8 | 6.4 |
|  | STD | 3.56 | 2.60 | 4.83 |

**PCL-5 Scores**

|  | **Group** | **Baseline** | **Week7** | **Week11** |
| --- | --- | --- | --- | --- |
| Control Group | Mean | 26.73 | 22.18 | 26.18 |
|  | STD | 18.58 | 19.71 | 21.30 |
|  |  |  |  |  |
| Intervention Group | Mean | 28.16 | 19.2 | 23.4 |
|  | STD | 24.30 | 19.07 | 19.76 |
